# Role of Stress as a Precipitating Factor of Creutzfeldt-Jakob Disease: A Case Series

**DOI:** 10.7759/cureus.98829

**Published:** 2025-12-09

**Authors:** Rita Relvas, Cassiana Vasconcelos, André Buinho, Nuno Monteiro, Sandra Castro Sousa

**Affiliations:** 1 Internal Medicine Department, Hospital de Cascais, Lisbon, PRT; 2 Psychiatry Department, Hospital de Cascais, Lisbon, PRT; 3 Neurology Department, Hospital de Cascais, Lisbon, PRT

**Keywords:** creutzfeldt-jakob disease, functional neurological disorder, neurodegenerative disease, psychiatry and neuroscience, transmissible spongiform encephalopathy

## Abstract

Easily mistaken for multiple neurological and psychiatric pathologies, Creutzfeldt-Jakob disease (CJD) is still underdiagnosed, with a difficult, late, and often retrospective recognition. Furthermore, its pathophysiology is poorly understood, and to date, no intervention has proven effective in its prevention or associated morbidity and mortality. The authors present a series of two clinical cases that not only expose its natural history and challenging differential diagnosis but also warn of a potential impact of psychological and stressful factors in its predisposition and/or precipitation.

## Introduction

Creutzfeldt-Jakob disease (CJD) is a rapidly progressive neurodegenerative disease with an underestimated incidence and inevitably fatal outcome, within one year in 70% of patients [1,2].

Being clinically heterogeneous, its neuropsychiatric symptoms, cerebellar manifestations, and pyramidal and extrapyramidal signs are commonly misjudged as other psychiatric and neurological conditions. It is their rapid progression and lack of response to interventions that raise suspicion. Diagnosis demands the exclusion of other etiologies and relies presumptively on clinical, imaging, electroencephalographic (EEG), and cerebrospinal fluid (CSF) analysis elements. Regardless, a conclusive diagnosis mandates histopathological confirmation in a postmortem brain biopsy. Nevertheless, mortality results from the absence of therapy capable of modifying disease progression, making treatment purely supportive.

The main obstacle to the development of efficient therapeutic tools is a currently incomplete understanding of CJD pathophysiology. There are three established categories of disease: sporadic, genetic, and acquired. The first one is both the most common (85%-95% of cases) and the least understood, its risk factors and potential triggers remaining so far mostly unknown [3,4]. In this variant, the possible role of psychological and stressful events has been advocated. Known as the stress hypothesis, this theory proposes that hypothalamic-pituitary-adrenal (HPA) axis pathways and stress-related inflammation may act as mediators in CJD pathophysiology. However, not only is there scarce evidence to support this theory, but also its interpretation is made difficult by the clinical manifestations of CJD, marked by the presence of psychiatric symptoms itself.

The case series here addressed provides further evidence on this topic, consistent with stress factors acting as CJD triggers. In addition, by reviewing and framing current knowledge on this topic, the authors went from clinical descriptions to a hypothesis-generating framework.

## Case presentation

Case 1

A 64-year-old woman with hypertension presented to the emergency room (ER) due to memory loss, gait imbalance, and behavioral changes (aggressiveness and delusions of persecution). The onset of symptoms, three months earlier, coincided with her husband’s health worsening. Being not only his wife but also his main caregiver in the context of Parkinson disease, his clinical deterioration was described as having had a large emotional impact on the patient, exacerbated by the lack of family or friends’ support. Deep sadness, despair, frustration, indignation, revolt, isolation, and depression were feelings reported by the patient. All of them got worse after her husband’s death, one month prior to the first visit to the hospital. At this first contact, on examination, she had a depressed mood (accounting 19 points in the Patient Health Questionnaire-9 {PHQ-9}), temporal-spatial disorientation, disorganized thoughts, hesitant speech, persecutory delusions, visual and auditory hallucinations, retrograde and episodic memory impairment (scoring 14 points in the Mini-Mental State Examination {MMSE}), insomnia, and gait imbalance, with small steps and wide base. A complete etiological study was performed, focused on excluding an infectious or immune process of the central nervous system, namely, autoimmune encephalitis. However, it revealed normal analytical results, as well as a normal CSF analysis. Also, cranial computed tomography (CT) and magnetic resonance imaging (MRI) showed chronic microangiopathic leukoencephalopathy, with diffusion restriction in the head and anterior segment of the body of the left caudate nucleus, considered nonspecific. Finally, the EEG excluded epileptiform activity. She was evaluated by neurology and psychiatry, and a probable functional disorder was assumed, superimposed on incipient vascular dementia. The patient was discharged with a follow-up office appointment.

**Figure 1:**
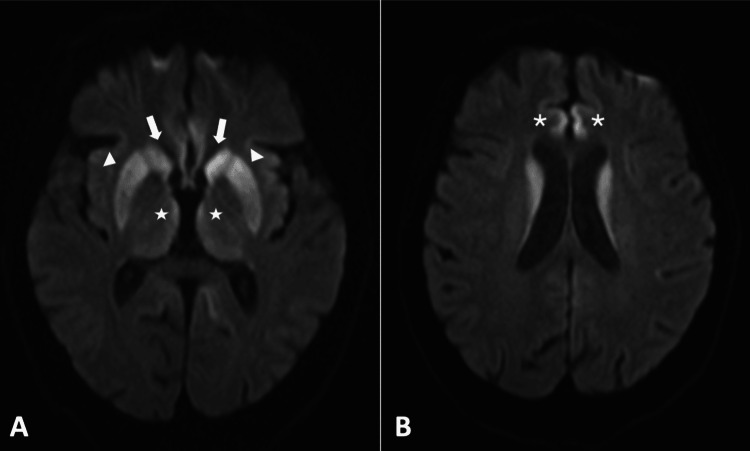
Neurodegenerative process associated with CJD: cranial MRI findings (Case 1). Subsequent axial cranial MRI diffusion-weighted imaging (DWI) sequence, showing restricted diffusion in both caudate nuclei (wide arrow), the putamen (arrowhead), internal and posterior surfaces of the thalami (star), and bilateral superior internal frontal gyrus (asterisk). CJD, Creutzfeldt-Jakob disease; MRI, magnetic resonance imaging

She returned one month later due to syncope. Thoracic CT angiography revealed pulmonary thromboembolism, promptly anticoagulated. However, a marked cognitive and motor deterioration stood out. At this point, the patient was accompanied by a neighbor, who had been taking care of her as she had become almost totally dependent since hospital discharge, both physically and cognitively. The neighbor described that during that one-month period, she had progressively gotten more apathetic, isolated and solitary, poorly communicative, and practically inactive. On clinical examination, in addition to the latter described, she presented inattention, poorly syntonic contact, facial hypomimia, psychomotor slowing, increased response latency, impoverished speech (monosyllabic and with poor content), emotional lability, and excessive suspiciousness. In addition, she had generalized muscular weakness, becoming practically unable to walk, as well as ataxia and bradykinesia. Not only was a thorough analytical study performed again, with innocent results, but also a second EEG was requested, showing moderate paroxysmal epileptic activity, with abrupt frontotemporal waves of left predominance. Another cranial MRI was performed, which showed restricted diffusion in the caudate nuclei, putamen, thalami, and frontal gyrus, highly suggestive of CJD (Figure 1A, 1B). To support this hypothesis, a second CSF study showed elevated protein 14.3.3 (>80000 AU/mL {normal range: <20000 AU/mL}). A presumptive diagnosis was made, and supportive measures were prioritized. The patient evolved unfavorably, with total mutism, generalized paresis, and permanent inactivity, with infectious complications and, ultimately, a fatal outcome after three weeks. A definitive diagnosis of sporadic CJD was histologically confirmed after postmortem brain biopsy.

Case 2

A 62-year-old woman with depression presented in a neurology office visit due to worsening depression, gait disturbance, memory complaints, language impairment, and autonomy loss. The symptoms began two months earlier, with onset coinciding with the diagnosis of severe maternal COVID-19 and family conflict. The patient described initial intrusive feelings of sadness, hopelessness, anxiety, fear, and anger related to the personal context, followed by a progressive ineptitude for her daily activities due to an inability to either concentrate or memorize and then an imbalance and speech disability. At this first visit, a depressive mood stood out, accounting for a score of 21 in the PHQ-9. Also, a mild cognitive impairment was perceived after applying MMSE (scoring 22 points), as well as a gait imbalance with no preferential deviation. A first cranial MRI was performed to rule out any structural cerebral disease. A normal result was obtained. Consequently, a diagnosis of functional disorder was assumed. Two weeks after this diagnosis, she presented to the ER due to worsening symptoms, namely, reduced walking ability, marked memory complaints, and total dependence. Clinically, the patient showed bizarre posture, stereotyped movements, scattered attention, temporal disorientation, perplexed mood, laconic speech, and response latency, as well as gait apraxia, hyperreflexia, and spasticity. Another MMSE was performed, with a marked downgrade score of 8. Basic blood analysis yielded normal results, with no elevation of systemic inflammatory markers nor the presence of substance use signs (in both blood and urine analyses). Also, a cranial CT angiography only revealed chronic ischemic leukoencephalopathy of small vessels. Even though an initial hypothesis of catatonic depression was made, the condition abruptly worsened within 48 hours, progressing to total mutism and refusal to eat, vertical nystagmus, myoclonus, wobbling, and postural changes, with the pronation of the forearms and the flexion of the wrists, culminating in coma and requiring admission to the intensive care unit (ICU).

**Figure 2:**
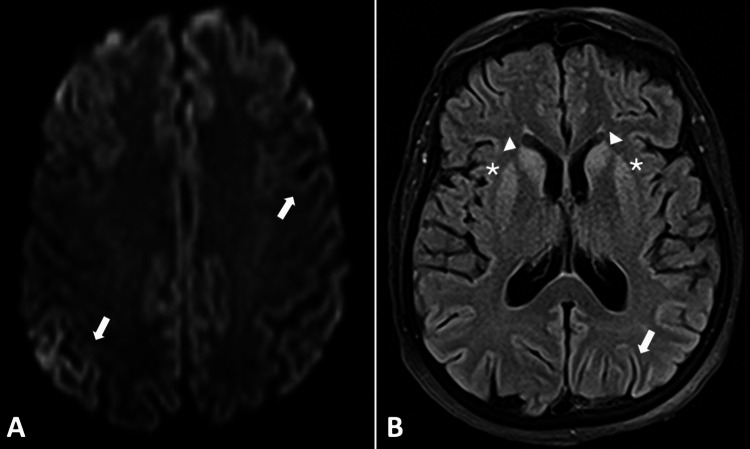
Neurodegenerative process associated with CJD: cranial MRI findings (Case 2). (A) Axial cranial MRI T2-FLAIR sequence, showing cortical ribbon sign (arrow). (B) Axial cranial MRI DWI sequence, showing both cortical ribboning (arrow) and hyperintensity of caudate nuclei heads (arrowhead) and the putamen (asterisk). CJD, Creutzfeldt-Jakob disease; FLAIR, fluid-attenuated inversion recovery; MRI, magnetic resonance imaging; DWI, diffusion-weighted imaging

At this stage, further investigation was obviously needed. An EEG showed periodic generalized discharges and criteria for non-convulsive status epilepticus, with levetiracetam 2000 mg twice daily and lacosamide 200 mg twice daily being introduced. However, paroxysmal activity persisted, even after the titration of levetiracetam and the introduction of sodium valproate 15 mg/kg/day. A repeated cranial CT scan showed new, nonspecific hypodense focal areas in the frontal thalamocapsular, mesencephalic, and protuberantia. Given the psychiatric-predominant onset, rapid progression, catatonic-like features, and non-convulsive status epilepticus, the hypothesis of an infectious or immune encephalitis was truly considered. Consequently, infectious and immune serology and lumbar puncture were performed, with normal results. Despite that, given the limitations of immune markers and the strong clinical suspicion of autoimmune encephalitis, a five-day cycle of intravenous immunoglobulin 0.4 mg/kg/day and methylprednisolone 1 g/day was started. Due to the lack of improvement and normal serum and CSF results, immunomodulatory therapy was suspended. A new cranial MRI was only possible a few days after ICU admission, two days after immunomodulatory therapy suspension. It showed signal alteration in T2-fluid-attenuated inversion recovery (FLAIR) and diffusion-weighted imaging (DWI) sequences, both in the cerebral cortex and striatum, suggestive of CJD (Figure 2A, 2B). Considering this hypothesis, a repeated lumbar puncture was performed, specifically looking for protein 14.3.3 levels, which were >80000 AU/mL, consistent with this diagnosis. Histological confirmation was made post-mortem, three weeks after admission, in its sporadic variant.

To properly compare both cases and their similarities, Table 1 summarizes the cases described, highlighting the main clinical, analytical, and imagological manifestations of CJD. Moreover, it sums up the main characteristics of the patients, highlighting their background and possible precipitating factors involved, emphasizing the role of psychologically meaningful events as potential triggers.

**Table 1:** Summary of the case series: clinical, laboratory, and imaging features. Ag, antigen; AMPA, α-amino-3-hydroxy-5-methyl-4-isoxazolepropionic acid; ANA, antinuclear antibody; CJD, Creutzfeldt-Jakob disease; CRP, C-reactive protein; dsDNA, double-stranded DNA viruses; GABA, gamma-aminobutyric acid; HIV, human immunodeficiency virus; NMDA, N-methyl-D-aspartate; PCT, procalcitonin; RNP, ribonucleoprotein; RPR, rapid plasma reagin; Sm, Smith antigen; TPHA, *Treponema pallidum* hemagglutination assay; TSH, thyroid-stimulating hormone; VDRL, Venereal Disease Research Laboratory test; PHQ-9, Patient Health Questionnaire-9; MMSE, Mini-Mental State Examination; Ig, immunoglobulin; NK, natural killer; EEG, electroencephalogram; DWI, diffusion-weighted imaging; FLAIR, fluid-attenuated inversion recovery

	Patient 1	Patient 2
Age, years	64	62
Sex	Female	Female
Medical history	Hypertension	Depression
Possible precipitating factor	Husband’s fatal illness	Serious maternal illness and family conflict
Related psychological features	Deep sadness, despair, frustration, indignation, revolt, isolation, and depression (PHQ-9: 19 points)	Sadness, hopelessness, anxiety, fear, and anger (PHQ-9: 21 points)
Presenting symptoms		
Psychiatric symptoms	Yes: dementia (MMSE: 14 points), disorientation, inattention, apathy, emotional lability, heteroaggressiveness, insomnia, and psychosis (delusional ideas and auditory and visual hallucinations)	Yes: dementia (MMSE: 8 points), disorientation, inattention, depression, and bizarre and stereotypical posture
Language impairment	Yes: response latency and mutism	Yes: response latency and mutism
Gait impairment	Yes: gait ataxia, small steps, and robotic	Yes: gait ataxia
Myoclonus	No	Yes
Cerebellar manifestations	Yes: ataxia	Yes: ataxia and nystagmus
Pyramidal signs	Yes: spasticity	Yes: spasticity and hyperreflexia
Extrapyramidal signs	Yes: bradykinesia and rigidity	Yes: rigidity
Analytical study		
General biochemistry	Normal: liver, kidney, and thyroid function; complete ionogram; blood alcohol level	Normal: liver, kidney, and thyroid function; complete ionogram; blood alcohol level
Urinary assays	Negative: benzodiazepines and opioids	Negative: benzodiazepines and opioids
Infectious study	Normal: leukogram, CRP, and PCT; HIV (Ag p24+anti-HIV1/2); RPR, VDRL, and TPHA	Normal: leukogram, CRP, and PCT; HIV (Ag p24+anti-HIV1/2); RPR and VDRL; anti-herpes simplex 1 and 2 IgM/IgG and anti-Herpes zoster
Immunological study	Normal: ANA, anti-dsDNA, anti-SSA/Ro and anti-SSB/La, anti-Sm, anti-RNP (U1 RNP), lupus anticoagulant, anti-thyroglobulin (Tg), anti-TSH receptor (TrAb), anti-microsomal peroxidase (TPO), anti-glutamate decarboxylase (GAD67), anti-Purkinje cell (Yo), anti-neuron (Tr, Hu, ANNA-1 and Ma2/Ta, and PNMA2), anti-neuron nucleus (CV2, CRMP5 and Ri, and ANNA-2), anti-amphiphysin, anti-NMDA receptor, anti-GABAb receptor, anti-phospholipid IgM and IgG, anti-AMPA receptor (GluR1 and GluR2) and anti-AMPA receptor glycine (GlyR), anti-CASPR2, and anti-LGI1	Normal: ANA, anti-dsDNA, anti-cardiolipin IgM and IgG, anti-beta-2 glycoprotein I IgM and IgG, lupus anticoagulant, anti-Tg, anti-TrAb, anti-TPO, anti-GAD67, anti-Yo, anti-neuron (Hu, ANNA-1 and Ma2/Ta, and PNMA2), anti-neuron nucleus (CV2, CRMP5 and Ri, and ANNA-2), anti-MAG IgM and IgG, anti-NMDA receptor, anti-GABAb receptor, anti-AMPA receptor (GluR1 and GluR2) and anti-GlyR, anti-CASPR2, anti-potassium channel (BGKC), and anti-LGI1
Cerebrospinal fluid study		
Cytochemical study	Normal	Normal
Cytological study	Normal	Normal
Microbiological study	Negative	Negative
Herpesviridae DNA	Negative	Negative
Immunological study	Normal: anti-GABA and AMPA receptors, anti-NMDA, anti-CASPR2, anti-LGI 1, anti-GAD and anti-GAD67, anti-GlyR, anti-MAG, anti-*Borrelia burgdorferi* IgM and IgG, VDRL, and anti-*Brucella* spp. IgM and IgG	Normal: anti-GABA and AMPA receptors, anti-NMDA, anti-CASPR2, anti-LGI 1, anti-GAD and anti-GAD67, anti-GlyR, anti-MAG, and anti-PNMA2
Protein electrophoresis	Normal	Normal
Immunophenotyping	No phenotypic expression of B and NK cells	No phenotypic expression of B and NK cells
Immunofixation	Absence of oligoclonal bands	Absence of oligoclonal bands
ECA assay	Normal	Not carried out
14.3.3 protein assay	>80000 AU/mL (normal value: <20000 AU/mL)	>80000 AU/mL (normal value: <20000 AU/mL)
Electroencephalographic	Moderate paroxysmal activity with left-predominant frontotemporal abrupt waves	First EEG: non-convulsive status epilepticus. Second EEG: burst-suppression pattern, with epileptiform activity in the bursts
Cranial MRI	Signal change in DWI and T2-FLAIR sequences of the caudate nuclei, putamen, thalami, and frontal gyrus	Signal change in DWI and T2-FLAIR sequences of the cerebral cortex (cortical ribbon sign), caudate nuclei, and putamen
Duration, months	5 (approximately)	3 (approximately)
Histological diagnosis	CJD: sporadic variant	CJD: sporadic variant

## Discussion

The two cases described provide a proper ground for the revision of clinical manifestations, findings, and natural history of CJD. Furthermore, they raise suspicion that psychological and stressful factors may play a role in the predisposition and/or precipitation of this disease.

Despite continuous scientific research, the fatal outcome of this disease persists as a reality due to the lack of treatment options. Nowadays, it is known that CJD results from the transformation of prion protein (PrPC) into infectious proteinaceous particles, also called prions (PrPSc), capable of self-replication and accumulation in brain parenchyma [5]. These protein aggregates acquire a stable β-sheet conformation with significant neurotoxic effect, leading to neuronal loss, the absence of an effective inflammatory response, and the vacuolization of neutrophils, which acquire a spongiform appearance [6,7]. These anomalous particles lead not only to CJD but also to other transmissible spongiform encephalopathies [5]. Yet, the origin of these pathological prion proteins differs according to the form of the disease: genetic/familial/hereditary, acquired/infectious (variant and iatrogenic), or sporadic [8].

If, in its genetic form, some mutations have already been identified involving genes encoding prion proteins, their acquired form has also been elucidated [9]. On the one hand, the variant form of CJD seems to arise from the infection of humans by a bovine prion strain [10]. On the other hand, its iatrogenic form is known to be related to some medical procedures, such as the transplantation of the pituitary hormones, dura mater, cornea, and blood products from infected donors or the use of contaminated neurosurgical instruments [11].

Regarding its sporadic presentation, the origin of the pathological prion protein remains not fully understood. Some genetic polymorphs have already been characterized, with emphasis on the prion protein gene (*PRNP*), particularly codons 129 and 219 and, with less evidence, syntaxin 6 (STX6) and galactose-3-O-sulfotransferase 1 (GAL3ST1) [12,13]. In fact, it is from these polymorphisms that the subdivision of the sporadic form arises, with some phenotypic particularities and larger involvement of specific brain areas [13]. Simultaneously, multiple epidemiological studies have tried to determine other possible risk factors. Although not well-founded and possibly biased by the heterogeneity of local surveillance, the possibility of disease clusters with a common external factor has been pointed out as well [14]. Some authors invoke the association with some surgical procedures not recognized as causing the iatrogenic form, predominantly in women and especially when it comes to skin stitches, nose and throat surgeries, and the removal of growths/cysts [15,16]. Furthermore, ear piercings, living in or near farms (especially for more than 10 years) or near livestock, health professionals, personal history of dementia or poliomyelitis, and family history of CJD were also risk factors listed [15,17].

By reanalyzing our case series, one may recognize a possible common precipitating factor that has not yet been fully explored. Specifically, in both cases, the onset of behavioral changes and cognitive impairment coincided in time with emotionally stressful events: the death of the patient’s husband (Case 1) and serious maternal illness and family conflict (Case 2). These situations undoubtedly led to an emotional vulnerability, where feelings of sadness, anxiety, anger, despair, frustration, indignation, revolt, isolation, and fear were initially recorded. To date, little has been investigated regarding the influence of mental health on the natural history of CJD, neither as a predisposing feature nor as a precipitating factor. The related literature is scarce, and its interpretation is made difficult by the clinical manifestations of CJD, which raises the question of whether those psychological features are a risk factor or already a symptom of incipient disease.

On the one hand, in what concerns to a possible predisposing treat, a study found, in patients with sporadic CJD, a higher incidence of personal history of psychiatric illness needing follow-up in psychiatric consultation compared to control group (without CJD), with a statistically significant association (odds ratio {OR}: 2.6; 95% confidence interval {CI}: 1.5-4.3) [16]. Similarly, a meta-analysis of three case-control studies has corroborated the presence of a statistically significant association between sporadic CDJ and a history of psychiatric illness, particularly psychotic illness (OR: 9.9; 95% CI: 1.1-86.1) [17]. Notably, in the first study mentioned, the association remained statistically significant after excluding patients with follow-up of less than two years, which, considering the average survival of patients since the onset of symptoms in CDJ, makes it less likely that this symptomatology is solely explained by this disease [16].

On the other hand, the possibility of a stressful experience acting as a trigger event to CJD is emphasized by our case series. In fact, this possibility was first raised in 1997, when a group of French investigators published a case-control study comparing patients with CJD to matched controls [18]. In fact, they have found a highly significant relationship between the occurrence of CJD and major life events occurring up to five years before CJD onset. However, they did not quantify the strength of this association, precluding any possible evaluation of this hypothesis. Later on, in 1999, a pilot case-control study was developed in Germany, aiming to test the association of CJD with stressful life events [19]. The authors found a significantly higher proportion of patients with CJD experiencing stressful life events during the last six months before disease onset when compared to controls (65% versus 32%, p = 0.01, with OR of 3.85 and 95% CI of 1.33±11.30). Both studies included CJD cases, not differentiating the disease’s variant (familial, acquired, or sporadic). In 2008, the role of stress and anxiety in the onset of the familial form of CJD was deepened, and some theories were proposed [20]. However, although this scattered evidence seemed to raise the question of the role of stress factors in triggering CDI, not only was this not explored in depth in all forms of CJD, but also it was not specifically described in its sporadic variant.

When trying to find some causality between stressful events and CJD, some proposals have already been postulated, aiming to better understand what is called the stress hypothesis. It is known that stressors of any kind (psychological, physical, or biological) impact on cells directly or indirectly, influencing intracellular parameters (such as pH or electrolytes) and leading to proteotoxic damage and protein denaturation. These denatured proteins tend to aggregate and precipitate cell damage [20]. It is believed that the trait feature of CJD is precisely the accumulation in the brain of an affected isoform of prion protein. Accordingly, it is first postulated that cellular stress increases the expression of both PrP mRNA and protein, among several other proteins. This upregulation may lead either to the loss of function of the natively folded PrPc or to the gain of a toxic activity of PrPSC, with the accumulation of misfolded proteins, thus leading to the pathogenesis of CJD [20]. Also, there is solid evidence on the importance of biochemical stress mediators on neuronal cell death, such as interleukins (IL) 1 and 6 and glucocorticoid hormones. Indeed, it was already shown that glucocorticoids enhance cell death in hippocampal neurons by the oxidative stressors amyloid beta-protein and glutamate. In fact, the HPA axis activation secondary to a stressful trigger leads to a glucocorticoid-mediated modulation of prion protein expression [19]. In addition, other stress-induced neuroinflammatory pathways may also contribute to this association. In fact, together with the knowledge that endogenous IL-1 may be a mediator of neuronal cell death, evidence that IL-1 and tumor necrosis factor (TNF) were increased in the brains of scrapie-inoculated mice points to these mediators as possible contributors [19]. Finally, microglial priming potential facilitation of misfolding or propagation under stress-related cellular conditions (e.g., oxidative stress, endoplasmic reticulum stress, and chaperone dysregulation) may also play a not despicable role [20].

## Conclusions

The described cases highlight the possible role of stressful events in the natural history of CJD, postulating that they may act as precipitating factors. The authors aim to emphasize the need for large-scale studies that allow for a better understanding of the risk factors behind this disease, particularly with regard to psychological factors. The work brings to the table several observations and considerations that we believe would be of great value to be further explored. First, we emphasize the need to better characterize, through systematic reviews, the stressors themselves, in terms of type, intensity, duration, and time relation with the disease onset. Also, we show that a deeper knowledge of HPA axis pathways and stress-related inflammatory mediators is required in CJD pathophysiology. Accordingly, it might be of great significance to explore some biomarkers that can predictably correlate stress burden with disease emergence or the rate of progression, such as specific inflammatory cytokines, neurofilament light chain (NfL), or the application of real-time quaking-induced conversion (RT-QuIC). A more thorough understanding of CJD pathophysiological mechanisms is crucial for the development of future prevention strategies, better approaches, and therapeutic interventions for this disease, critically improving its clinical course.
